# Mid-trial dosimetric analysis of the korean radiation oncology group 19 − 09 multi-institutional prospective cohort study on breast cancer

**DOI:** 10.1186/s13014-026-02904-y

**Published:** 2026-08-11

**Authors:** Wonhyeong Lee, Yeon-Joo Kim, Tae Hyeon Kim, Won Park, Won Kyung Cho, Nalee Kim, Jong Hoon Lee, Sung-Ja Ahn, Jin Hee Kim, Mi Young Kim, Jongmoo Park, Yoonsun Chung

**Affiliations:** 1 Department of Radiation Oncology, National Cancer Center, Goyang, Republic of Korea; 2 Department of Nuclear Engineering, Hanyang University, Seoul, Republic of Korea; 3 Department of Radiation Oncology, Samsung Medical Center, Sungkyunkwan University School of Medicine, Seoul, Republic of Korea; 4 Department of Radiation Oncology, St. Vincent’s Hospital, The Catholic University of Korea College of Medicine, Seoul, Republic of Korea; 5 Department of Radiation Oncology, Chonnam National University Medical School, Gwangju, Republic of Korea; 6 Department of Radiation Oncology, Keimyung University Dongsan Hospital, Daegu, Republic of Korea; 7 Department of Radiation Oncology, Kyungpook National University Chilgok Hospital, Daegu, Republic of Korea; 8 Department of Radiation Oncology, School of Medicine, Kyungpook National University, Daegu, Republic of Korea

**Keywords:** Breast cancer, Regional nodal irradiation, Neoadjuvant chemotherapy, Artificial Intelligence, Auto-contouring

## Abstract

**Background:**

The Korean Radiation Oncology Group 19 − 09 multi-institutional clinical trial investigates the effect of regional nodal irradiation (RNI) omission on regional recurrence rates after neoadjuvant chemotherapy followed by breast-conserving surgery, and sentinel lymph node biopsy in cT1-3N1 and ypN0 breast cancer patients. This mid-trial analysis evaluates dosimetric deviations among enrolled patients using an artificial intelligence (AI) auto-contouring engine.

**Methods:**

We analyzed dose-volume histograms of 366 patients enrolled from eight institutions based on an AI auto-contoured reference structure set. Of these, 176 received whole breast and regional nodal irradiation (WBI + RNI), while 190 received whole breast irradiation only (WBI).

**Results:**

Dosimetric outliers were observed in both groups. In the WBI + RNI group, 25 (14%) and 30 (17%) patients were outliers in D95% of axillary levels II and III, respectively. The corresponding counts in the WBI group were 17 (9%) and 16 (8%), respectively. Especially, 7 (58%) of 12 patients treated by one physician in the WBI + RNI group were outliers for axillary level III. Similarly, among the 49 patients treated by another physician in the WBI + RNI group, 21 (43%) were outliers for axillary level II and 21 (43%) for level III. Regarding the breast CTV, all outlier patients except one were treated by the two physicians.

**Conclusion:**

Significant dose deviations were observed, indicating that comparing the WBI + RNI and WBI groups alone may inadequately assess the clinical impact of RNI omission. Therefore, the final analysis will evaluate the association between specific regional nodal doses and clinical outcomes.

**Supplementary Information:**

The online version contains supplementary material available at https://doi.org/10.1186/s13014-026-02904-y.

## Introduction

Previous studies have reported that patients with clinical T1-3N1 breast cancer who undergo neoadjuvant chemotherapy (NAC) followed by breast-conserving surgery (BCS) and achieve no residual nodal disease (ypN0) have a very low rate of lymph node (LN) recurrence even when regional nodal irradiation (RNI) is omitted [1–3]. However, there remains no clear evidence supporting the RNI omission in such patients [4], and the National Comprehensive Cancer Network (NCCN) guideline also recommends radiotherapy (RT) based on the worst clinical stage [5]. Furthermore, the previous retrospective studies evaluating RNI omission have primarily included patients who underwent axillary lymph node dissection (ALND) [2, 3]. Sentinel lymph node biopsy (SLNB) has largely replaced invasive ALND after NAC, based on studies demonstrating its acceptable false-negative rate [6–8]. Although the National Surgical Adjuvant Breast and Bowel Project (NSABP) B-51 phase III clinical trial reported comparable 5-year clinical outcomes regardless of RNI in ypN0 patients following NAC [9], it included both ALND and SLNB, without specifically focusing on patients treated with SLNB. In the absence of prospective data on the safety of omitting RNI in ypN0 patients undergoing SLNB, the Korean Radiation Oncology Group (KROG) initiated a prospective cohort study to evaluate the effect of RNI omission on the regional recurrence rate after NAC followed by BCS and SLNB in clinical T1-3 with LN metastasis in axillary (Axl) level I and ypN0 breast cancer patients (KROG 19 − 09, IRB No. NCC2019-0279; CRIS No. KCT0004567) in October 2019. Enrolled patients were assigned to two groups through a shared decision-making process (Additional File 1).

Actual delivered dose to the regional nodal areas, rather than the prescribed dose, impacts regional recurrence and other clinical outcomes. However, a retrospective study reported that substantial protocol deviations in RNI plans may compromise clinical outcomes in multi-institutional trials [10]. Considering that KROG 19 − 09 is a prospective, multi-institutional cohort study, similar or even greater deviations in RNI plans could be anticipated. In response to concerns about the potential impact of these deviations on clinical outcomes, a dummy run quality assurance (QA) for KROG 19 − 09 was conducted before expanding to a multi-institutional study [11]. However, a review of radiotherapy quality assurance (RTQA) practices emphasized that pre-trial QA alone does not ensure consistency in RT plans, underscoring the importance of follow-up review after patient enrollment begins [12].

Since commercial artificial intelligence (AI) auto-contouring engines in planning computed tomography (CT) for breast cancer RT became available after 2022 [13], the incorporation of an AI auto-contouring engine was considered for KROG 19 − 09 [11]. Anticipating that AI auto-contoured structures could reduce inter-institutional dosimetric deviations in RT planning, the host institution developed a customized version of a commercial AI auto-contouring engine (AVIEW, Coreline Soft Inc., Seoul, Korea) for the KROG 19 − 09 study. This engine was distributed to participating institutions in April 2024. In the final analysis, we will compare deviations before and after AI implementation.

We conducted a mid-trial analysis to monitor institutional dosimetric deviations within ongoing KROG 19 − 09. This study mainly analyzed dose-volume histograms (DVHs) of patients treated before the introduction of the AI auto-contouring engine. The primary objective is to identify critical deviations that need to be addressed and provide insights into evaluation strategies for the final analysis. Crucially, this mid-trial analysis also serves as a proactive quality intervention. By sharing the interim findings and introducing the AI auto-contouring engine, we aim to ensure dosimetric consistency for the reliability of the trial.

## Materials & Methods

### Subjects

**Fig. 1:**
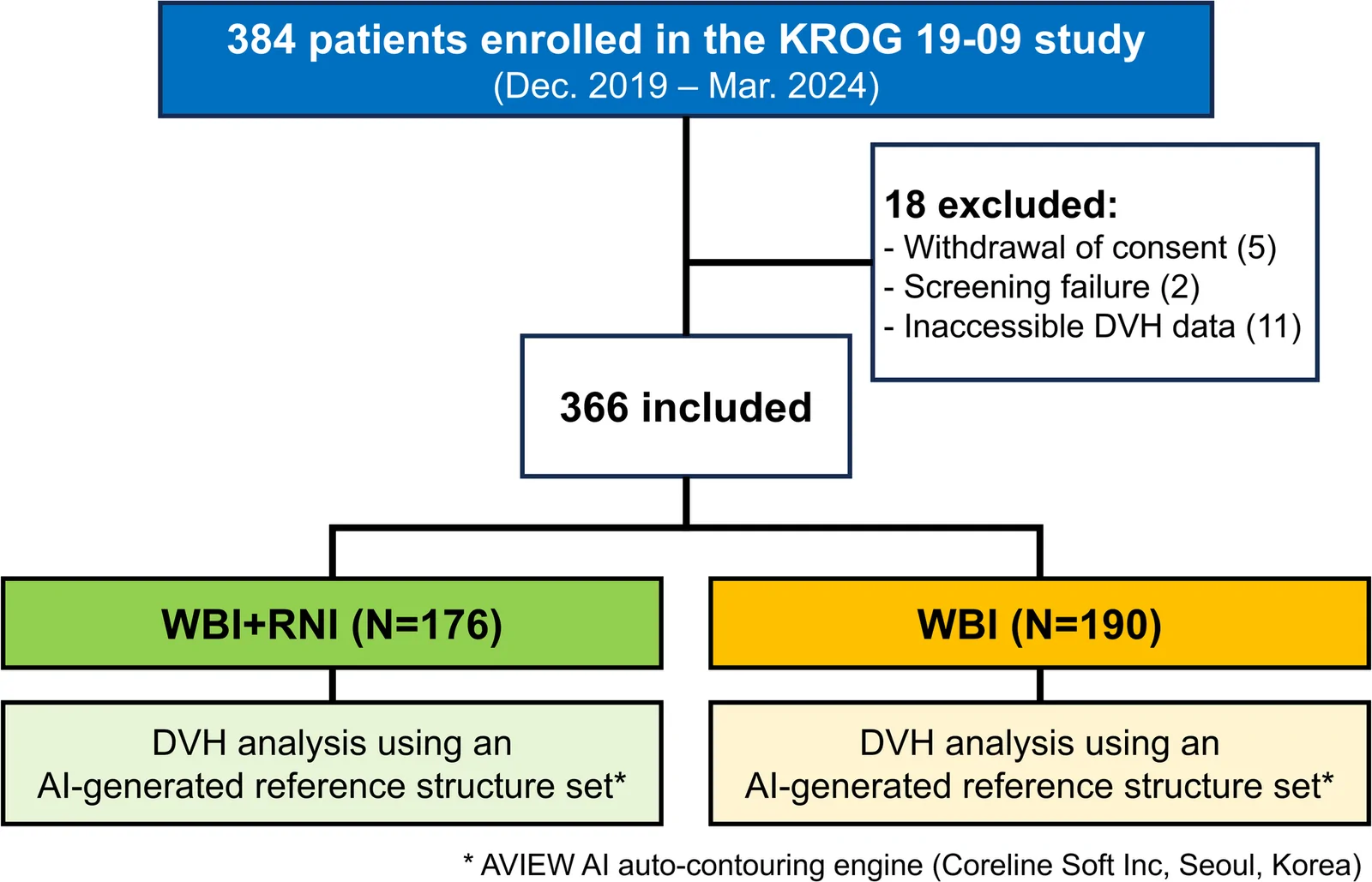
Flowchart of the mid-trial analysis in the KROG 19 − 09 study. Abbreviations: DVH, dose-volume histogram; WBI, whole breast irradiation; RNI, regional nodal irradiation

Figure 1 illustrates the flowchart of this mid-trial analysis. Before the implementation of the AI auto-contouring engine, 384 of the target 844 patients (45%) were enrolled for KROG 19 − 09 between December 2019 and March 2024. Ten physicians from eight institutions planned treatment for these patients according to the KROG 19 − 09 protocol and institutional policies. Eighteen patients were excluded from this analysis for the following reasons: five for withdrawal of consent, two for screening failure, and eleven for inaccessible DVH data. Among the 366 patients included in this mid-trial analysis, 176 received both whole breast and regional nodal irradiation (WBI + RNI group), while 190 received whole breast irradiation only (WBI group). The group allocation followed the treating physician’s intent; the WBI + RNI group included patients who received RT to Axl levels I-II only, without Axl III or supraclavicular lymph node (SCN) coverage. Internal mammary node (IMN) inclusion was also at the treating physician’s discretion.

### Data analysis

The host institution received anonymized Digital Imaging and Communications in Medicine (DICOM) files of enrolled patients from participating institutions and analyzed the DVHs. A reference structure set for DVH extraction was generated using the customized AI auto-contouring engine. Building upon a validated commercial baseline engine [14–16], we optimized the algorithm to delineate additional structures and accommodate diverse patient postures, adhering to the contouring recommendations of the KROG 19 − 09 protocol [11]. For clinical target volumes (CTVs), the minimum dose delivered to 95% volume of the structure (D95%) relative to the prescribed dose was calculated. Breast and the following regional nodal areas were included as reference CTV structures: Axl levels I, II, and III, interpectoral LN, both SCN based on the European Society for Radiotherapy and Oncology (ESTRO) and the Radiation Therapy Oncology Group (RTOG) consensus guidelines [17, 18], and IMN. For organs at risk (OARs), the following DVH parameters were extracted from the reference structure set: mean heart dose, volume receiving 20 Gy (V20Gy) for the ipsilateral lung, volume receiving 5 Gy (V5Gy) for the contralateral breast, and volume receiving 15 Gy (V15Gy) for the ipsilateral shoulder joint.

The extracted DVH parameters were presented as median (range). Differences in median DVH parameters between the WBI + RNI and WBI groups were evaluated using the Mann-Whitney U test. Boxplots and histograms were used to visualize dosimetric distributions. The boxplots display the minimum, maximum, first quartile (Q1), third quartile (Q3), and median values across all patients. Outliers were defined as values exceeding 1.5 times the interquartile range (IQR = Q3 – Q1) beyond Q1 or Q3 and were plotted as dots in the boxplots. The histograms depicted the frequency distribution across intervals for each DVH parameter.

In both groups, we calculated the proportion of outliers in the D95% values. This was obtained for all patients within the groups and 10 physicians each (labeled in A-J) to evaluate dosimetric deviation by physician level. We selected representative RT plans from each group to compare RT plans among physicians. The selected RT plans were visualized using Varian’s Eclipse™ treatment planning system, with beam’s eye view displays of the 95% isodose line.

### Statistical analysis

All statistical analyses were performed using Python (version 3.12). We used the Matplotlib (version 3.8.2) library in generating boxplots and histograms, and the Scipy (version 1.13.1) library in performing the Mann-Whitney U test. Any p-value less than 0.05 was considered statistically significant.

## Results

### Patient, tumor, and treatment characteristics

Table 1 summarizes the patient, tumor, and treatment characteristics of the enrolled 366 patients. The median number of LNs harvested in SLNB was 2 (range, 0–14). Among the patients, 184 (50.3%) achieved a pathologic complete response (ypCR) after neoadjuvant therapy. A total of 146 patients (39.9%) received three-dimensional conformal RT (3D-CRT), while 220 (60.1%) received intensity-modulated RT (IMRT). All 366 patients underwent NAC, and 75 (20.5%) received adjuvant chemotherapy after surgery. Additionally, 172 (47.0%) patients received hormonal therapy, and 159 (43.4%) received anti-human epidermal growth factor receptor 2 (HER2) therapy.

**Table 1:** Patient, tumor, and treatment characteristics (*n* = 366)

	*n* (%)
Age (y)	
Median (range)	50 (23–76)
**Pathology**	
Invasive ductal carcinoma	356 (97.3%)
Invasive lobular carcinoma	6 (1.6%)
Others	4 (1.1%)
**Histological grade**	
1	11 (3.0%)
2	111 (30.3%)
3	194 (53.0%)
N/A	50 (13.7%)
**Tumor size on the MRI image (cm)**	
≤2.0	62 (16.9%)
2.1-5.0	268 (73.2%)
>5.0	36 (9.8%)
Median (range)	2.9 (0–10)
**Number of axillary level I lymph nodes on the MRI image**	
≤3	336 (91.8%)
>3	29 (7.9%)
N/A	1 (0.3%)
Median (range)	2 (1–11)
**Clinical tumor subtype**	
Luminal A-like (HR+/HER2- and Ki-67 < 20%)	12 (3.3%)
Luminal B-like (HR+/HER2- and Ki-67 ≥ 20%; HR+/HER2+)	151 (41.3%)
HER2 type (HR-/HER2+)	72 (19.7%)
Triple-negative (HR-/HER2-)	120 (32.8%)
N/A	11 (3.0%)
**Breast**	
Left	187 (51.1%)
Right	179 (48.9%)
**Surgical type of lymph node**	
SLNB only	222 (60.7%)
SLNB + axillary sampling	142 (38.8%)
N/A	2 (0.5%)
**Number of harvested sentinel lymph nodes**	
0	16 (4.4%)
1–3	256 (69.9%)
≥4	92 (25.1%)
N/A	2 (0.5%)
Median (range)	2 (0–14)
**ypCR**	
Yes	184 (50.3%)
No	182 (49.7%)
**ypN stage**	
pN0	362 (98.9%)
pN0 (i+)	4 (1.1%)
**Resection margin** (*n* = 182)	
Negative	171 (94.0%)
Positive	5 (2.7%)
N/A	6 (3.3%)
**Radiation therapy**	
3D-CRT	146 (39.9%)
IMRT	220 (60.1%)
**Neo-adjuvant chemotherapy**	
Yes	366 (100.0%)
No	0 (0%)
**Adjuvant chemotherapy after surgery**	
Yes	75 (20.5%)
No	291 (79.5%)
**Hormonal therapy**	
Yes	172 (47.0%)
No	194 (53.0%)
**Anti-HER2 therapy**	
Yes	159 (43.4%)
No	207 (56.6%)
**Inclusion of SCN irradiation in the WBI + RNI group** (*n* = 176)	
Yes	142 (80.7%)
No	34 (19.3%)
**Inclusion of IMN irradiation in the WBI + RNI group **(*n* = 176)	
Yes	14 (8.0%)
No	162 (92.0%)

*Abbreviations*: MRI, magnetic resonance imaging; HR, hormone receptor; HER-2, human epidermal growth factor receptor 2; SLNB, sentinal lymph node biopsy; ypCR, pathologic complete response after neoadjuvant therapy; 3D-CRT, three-dimensional conformal radiation therapy; IMRT, intensity-modulated radiation therapy; SCN, supraclavicular lymph node; WBI, whole breast irradiation; RNI, regional nodal irradiation; IMN, internal mammary lymph node

### DVH Comparisons

**Fig. 2:**
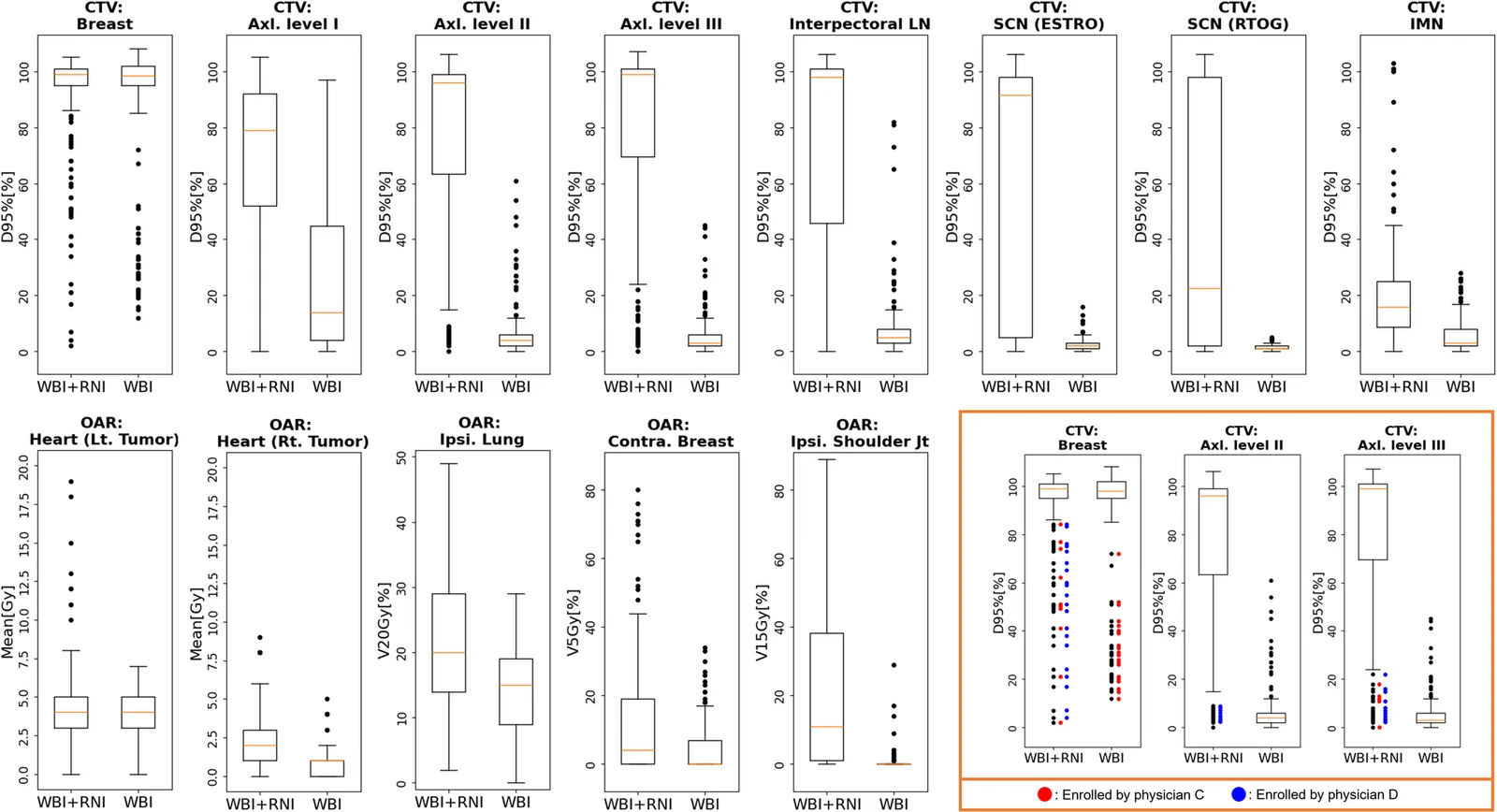
Boxplots of DVH distributions in the WBI + RNI and WBI groups. The orange-colored box shows outliers enrolled by specific physicians. Abbreviations: WBI, whole breast irradiation; RNI, regional nodal irradiation; CTV, clinical target volume; Axl, axillary; LN, lymph node; SCN, supraclavicular node; ESTRO, European Society for Radiotherapy and Oncology; RTOG, Radiation Therapy Oncology Group; IMN, internal mammary node; D95%, minimum dose delivered to 95% volume of the structure relative to the prescribed dose; OAR, organ at risk; Lt, left; Rt, right; Ipsi, ipsilateral; Contra, contralateral; Shoulder Jt, shoulder joint; V20Gy, volume receiving 20 Gy; V5Gy, volume receiving 5 Gy; V15Gy, volume receiving 15 Gy; DVH, dose-volume histogram

Table 2 compares the DVH parameters of CTVs and OARs between the WBI + RNI and WBI groups. All regional nodal areas demonstrated statistically significant differences (p-value < 0.01) in median D95% between the two groups, whereas no significant difference was observed for the breast CTV (p-value = 0.23). Similarly, all OAR-related DVH parameters differed significantly between the groups (p-value < 0.01), except for the mean heart dose in patients with left-sided tumors (p-value = 0.97). Eight patients with left-sided tumors received mean heart doses ranging from 10 Gy to 19 Gy, and seven patients with right-sided tumors received doses ranging from 5 Gy to 9 Gy. These distributions are also presented as histograms in Additional File 2. Despite these findings, boxplots in Fig. 2 reveal dosimetric outliers in the CTVs of both breast and regional nodal areas across both groups. The orange-colored box in Fig. 2 highlights that most of the outliers in breast CTV and Axl levels II and III were associated with specific physicians (Physicians C and D).

**Table 2:** Comparison of dose-volume histogram parameters in the WBI + RNI and WBI groups

				Median (range)		*p-value*
				WBI + RNI group (*N* = 176)	WBI group (*N* = 190)	
CTV	Breast		D95% [%]	99 (2-105)	98 (12–108)	0.23
	Axl. level I		D95% [%]	79 (0-105)	14 (0–93)	< 0.01
	Axl. level II		D95% [%]	96 (0-106)	4 (0–61)	< 0.01
	Axl. level III		D95% [%]	99 (0-107)	3 (0–45)	< 0.01
	Interpectoral LN		D95% [%]	98 (0-106)	5 (0–82)	< 0.01
	SCN (ESTRO)		D95% [%]	92 (0-106)	2 (0–16)	< 0.01
	SCN (RTOG)		D95% [%]	23 (0-106)	1 (0–5)	< 0.01
	IMN		D95% [%]	16 (0-103)	3 (0–28)	< 0.01
OAR	Heart	Left-sided Tumor	Mean [Gy]	4 (0–19) (*N* = 95)	4 (0–7) (*N* = 92)	0.97
		Right-sided Tumor	Mean [Gy]	2 (0–9) (*N* = 81)	1 (0–5) (*N* = 98)	< 0.01
	Ipsilateral Lung		V20Gy [%]	20 (2–49)	15 (0–29)	< 0.01
	Contralateral Breast		V5Gy [%]	4 (0–80)	0 (0–34)	< 0.01
	Ipsilateral Shoulder Joint		V15Gy [%]	12 (0–89)	0 (0–29)	< 0.01

*Abbreviations*: WBI, whole breast irradiation; RNI, regional nodal irradiation; CTV, clinical target volume; Axl, axillary; LN, lymph node; SCN, supraclavicular node; ESTRO, European Society for Radiotherapy and Oncology; RTOG, Radiation Therapy Oncology Group; IMN, internal mammary node; D95%, minimum dose delivered to 95% volume of the structure relative to the prescribed dose; OAR, organ at risk; V20Gy, volume receiving 20 Gy; V5Gy, volume receiving 5 Gy; V15Gy, volume receiving 15 Gy

## Outlier analysis by physician

Table 3 details the overall and physician-specific outlier rates for CTVs in both groups. For the breast CTV, 34 (19%) and 40 (21%) patients were outliers in the WBI + RNI and WBI groups, respectively. Especially, all 12 and 39 patients treated by Physician C in each group, and 22 (45%) patients treated by Physician D in the WBI + RNI group, were outliers for the breast CTV. For the regional nodal areas, 7 (58%) patients treated by Physician C were outliers for Axl level III in the WBI + RNI group. Among the patients treated by Physician D in the WBI + RNI group, 21 (43%) were outliers for Axl level II and 21 (43%) for level III. Additional File 3 summarizes the number of cases failing to meet the clinical dose thresholds (D95% < 90% of the prescribed dose) for each regional nodal area.

**Table 3:** Outlier counts for D95% of CTVs by physician

		Breast	Axl. level I	Axl. level II	Axl. level III	Interpectoral LN	SCN (ESTRO)	SCN (RTOG)	IMN
**Sum**	**WBI + RNI** (*N* = 176)	34 (19%)		25 (14%)	30 (17%)				11 (6%)
	**WBI** (*N* = 190)	40 (21%)		17 (9%)	16 (8%)	17 (9%)	7 (4%)	15 (8%)	11 (6%)
**A**	**WBI + RNI** (*N* = 33)								1 (3%)
	**WBI** (*N* = 83)	1 (1%)		9 (11%)	8 (10%)	9 (11%)	6 (7%)	15 (18%)	10 (12%)
**B**	**WBI + RNI **(*N* = 17)			1 (6%)					
	**WBI **(*N* = 62)			2 (3%)	1 (2%)	2 (3%)	1 (2%)		1 (2%)
**C**	**WBI + RNI** (*N* = 12)	12 (100%)		1 (8%)	7 (58%)				
	**WBI** (*N* = 39)	39 (100%)		5 (13%)	6 (15%)	5 (13%)			
**D**	**WBI + RNI** (*N* = 49)	22 (45%)		21 (43%)	21 (43%)				5 (10%)
	**WBI** (*N* = 0)								
**E**	**WBI + RNI** (*N* = 29)			1 (3%)	1 (3%)				5 (17%)
	**WBI** (*N* = 2)			1 (50%)	1 (50%)	1 (50%)			
**F**	**WBI + RNI** (*N* = 14)			1 (7%)					
	**WBI** (*N* = 3)								
**G**	**WBI + RNI** (*N* = 9)								
	**WBI**(*N* = 0)								
**H**	**WBI + RNI** (*N* = 9)								
	**WBI** (*N* = 0)								
**I**	**WBI + RNI**(*N* = 3)								
	**WBI** (*N* = 0)								
**J**	**WBI + RNI** (*N* = 1)								
	**WBI** (*N* = 1)								

*Abbreviations*: D95%, minimum dose delivered to 95% volume of the structure relative to the prescribed dose; CTV, clinical target volume; WBI, whole breast irradiation; RNI, regional nodal irradiation; Axl, axillary; LN, lymph node; SCN, supraclavicular node; ESTRO, European Society for Radiotherapy and Oncology; RTOG, Radiation Therapy Oncology Group; IMN, internal mammary node

**Fig. 3:**
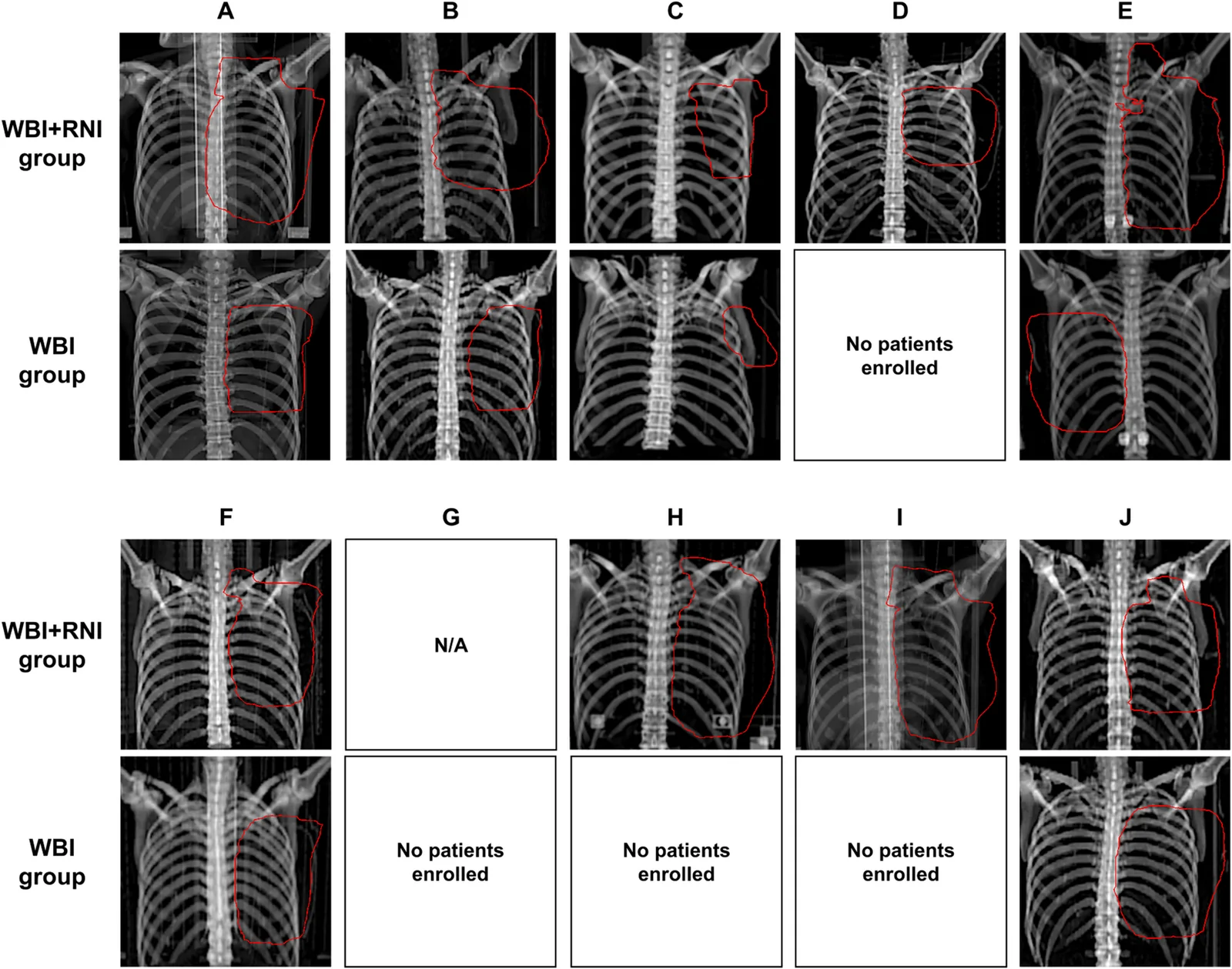
95% isodose lines (red line) for each physician in WBI + RNI and WBI groups. Abbreviations: WBI, whole breast irradiation; RNI, regional nodal irradiation

Figure 3 illustrates representative RT plans for patients in both groups by physicians, annotated with the 95% isodose line (red). For RT plans from Physician C, 95% isodose lines covered only a portion of breast in both groups. Some WBI + RNI plans by Physicians C and D covered only Axl levels I and II, excluding Axl level III and SCN from the RNI field.

## Discussion

We observed significant dosimetric deviations for patients enrolled in KROG 19 − 09 through a mid-trial analysis. Dosimetric QA for regional nodal areas is rarely performed in phase III breast cancer trials, even for limited subregions or selected patients [19–22]. The novelty of this study lies in its quantitative dosimetric analysis of all regional nodal areas across all enrolled patients. We found that higher doses were delivered to regional nodal areas in the WBI + RNI group. However, the presence of outliers—underdosing in the WBI + RNI group or overdosing in the WBI group—resulted in a broad, spectral distribution of doses across the regional nodal areas. Physicians C and D varied the target coverage of regional nodal areas across patients. For some patients, the treatment plans comprehensively included all LNs up to the SCN. Conversely, for other patients, the high-tangent technique was adopted to deliver the RNI dose. By raising the cranial border of the medial tangential field to within 2 cm of the humeral head [23], these plans included only Axl level I and a portion of level II. Consequently, a higher prevalence of outliers at Axl levels II and III was observed among WBI + RNI group patients treated by these two physicians. This indicates that the dosimetric deviations in regional nodal areas may be physician-dependent. For the SCN, although no outliers were observed in the WBI + RNI group, the boxplots revealed a wide D95% range (0-106%), attributable to inconsistent SCN inclusion in RNI.

Following trials reporting RNI benefits, Ling et al. established a separate network to investigate inter-institutional RNI plan deviations to assess its applicability at a clinical practice level [10]. They observed deviations in RNI plans and concluded that low compliance in the RNI plans potentially compromises the clinical benefit demonstrated in the previous trials. Although studies reporting dosimetric deviations in IMRT-based multi-institutional trials for breast cancer are limited, comparable levels of deviations have been observed in trials for other cancer types. In the Japan Clinical Oncology Group (JCOG) 1402 phase III trial of cervical cancer, the authors compared protocol compliance between pre- and post-enrollment RT plans. Although the average number of deviations per plan decreased from 3.1 to 1.4, significant target deviations remained. Notably, they identified repeated deviations from a single institution, resembling the physician-dependent patterns observed in our study [24]. In the RTOG 0933 phase II trial on hippocampal avoidance during whole-brain RT, pre-enrollment credentialing revealed 7–9% deviation rates. However, institutions exempt from pretreatment review had a 24% rate of unacceptable deviations in post-treatment review [25]. These findings demonstrate that protocols in clinical trials may diverge from clinical practice, suggesting the possibility that such a protocol may insufficiently reflect the clinical trial itself. Our mid-trial analysis revealed non-negligible dosimetric deviations in both WBI + RNI and WBI groups. Consequently, the current group allocations may not accurately represent the intended groups. This discrepancy potentially confounds the evaluation regarding the clinical impact of the RNI omission, the primary goal of the KROG 19 − 09 study. To overcome this issue, we will directly evaluate the association between specific regional nodal doses and clinical outcomes, alongside the analyses based on the current allocation.

The NSABP B-51 phase III clinical trial evaluated the benefit of RNI in 1,641 breast cancer patients with ypN0 after NAC, reporting no significant effect of RNI on recurrence or survival. However, the trial included patients treated with either ALND or SLNB between 2013 and 2020 [9]. In contrast, KROG 19 − 09 exclusively includes patients treated with SLNB since 2019. This distinction may provide clinical outcomes more reflective of SLNB and contemporary systemic therapy. Moreover, KROG 19 − 09 has enabled access to RT DICOM data of enrolled patients from participating institutions. For patients with regional recurrence, these DICOM files will allow a direct analysis of the delivered dose to the corresponding region. This supports accurate evaluation of the dose-response relationship within the KROG 19 − 09 study.

The previous KROG 17 − 01 dummy run demonstrated higher regional nodal doses in the WBI + RNI group than in the WBI group [26], consistent with findings from the KROG 19 − 09 dummy run [11]. Nevertheless, deviations in dose delivery were still observed in these studies—unintentional doses to Axl level I in the WBI group and insufficient doses to interpectoral LN and SCN in the WBI + RNI group. Similar patterns were observed in the present study, with a broad dose distribution and multiple outliers across the regional nodal areas. Meanwhile, KROG 08 − 06 reported substantial unintentional IMN doses in patients who were not prescribed IMN irradiation, both in dummy run and subsequent individual case review [20, 27]. The KROG 19 − 09 dummy run showed a reduced unintentional dose to the IMN, with a median D95% of 3.6–4.1% in the WBI group without IMN intent [11]. The current mid-trial analysis demonstrated a consistent trend, with a median IMN D95% of 3% under the same conditions.

We observed generally higher OAR doses in the WBI + RNI group, consistent with findings from the KROG 19 − 09 dummy run [11]. For the shoulder joint, the Standardisation of Breast Radiotherapy (START) pilot study reported that the RNI schedule and the equivalent dose in 2 Gy fractions (EQD2) influenced the estimated 5-year cumulative incidence of shoulder stiffness. The incidence ranged from 12% with schedules of 50 Gy/25 fx or 39 Gy/13 fx (46.1 Gy_3.5_ EQD2) to 33% with a schedule of 42.9 Gy/13 fx (53.0 Gy_3.5_ EQD2) [28]. This underscores the importance of shoulder joint dose considerations in RNI planning. However, the clinical impact of the shoulder joint dose from RNI has not been well investigated [29]. In the final analysis, we plan to evaluate the effect of RNI on shoulder stiffness based on the collected shoulder joint dose data.

Regarding mean heart dose, eight patients with left-sided tumors exceeded 10 Gy (including two IMN cases at 18 and 19 Gy). Similarly, seven patients with right-sided tumors exceeded 5 Gy (including three IMN cases at 8, 8, and 9 Gy). Physician E treated 14 of these 15 patients, suggesting a potential association between physician practice patterns and OAR dose deviation. A subsequent review revealed that the use of a chest thermoplastic mask for treatment reproducibility limited the physical separation between the heart and the chest wall target. This physical restriction, combined with the strict target coverage (V100% of planning target volume > 98% of the prescribed dose), resulted in elevated cardiac doses. To appropriately manage the associated risks, these patients will be highlighted for long-term cardiac event monitoring in the final analysis.

Recent breast cancer studies have demonstrated the robust performance of AI auto-contouring for breast CTV and OARs, while it remains challenging for regional nodal areas [13, 30]. Although the commercial baseline engine has been well-validated [14–16], the customized version used as a reference structure set in this study was limited by the lack of additional validation and confirmation by all participating physicians. Nonetheless, in all WBI + RNI group patients treated at the host institution, the D95% values were 87% or higher for Axl levels II, III, and SCN (ESTRO). This indirectly demonstrates acceptable conformity to the AI auto-contoured structures. For the final analysis, we will evaluate the changes in dosimetric deviation before and after the provision of the AI auto-contouring engine. Therefore, this mid-trial analysis includes only patients enrolled before its provision. Participating institutions have been able to apply the AI auto-contouring engine to patients enrolled since April 2024. Although institutions have the option to modify or disregard the AI auto-contoured structures, we will evaluate their potential contribution to reducing dosimetric deviations in the final analysis.

The host institution shared the findings of this mid-trial analysis with participating institutions through periodic joint meetings and discussed the future direction of the KROG 19 − 09 study. Regarding the breast CTV, all outlier patients except one were treated by Physicians C and D. This was primarily attributable to the use of partial breast irradiation (PBI) in their RT plans, as illustrated in Fig. 3. Since PBI is deemed ineligible for this study, which consists of patients with initial node-positive status receiving NAC [31], all institutions agreed to transition from PBI to WBI for future patients to better align with the study design. To appropriately handle the 36 patients already treated with PBI before the mid-trial analysis, our final analysis will either exclude them from the primary evaluation or assess them within a subgroup. Meanwhile, the original protocol specified that patients who received SCN irradiation should be assigned to the WBI + RNI group. However, deviations in RT planning were observed among some physicians who applied a high tangent technique concept for RNI, which does not irradiate SCN. After consensus, we broadened the assignment criteria for the WBI + RNI group to also include RNI plans using the high tangent technique. The 366 patients in this mid-trial analysis followed this revised grouping, which will be maintained for future patient enrollment. In the final analysis, exploratory subgroup analyses stratified by delivered dose will be conducted, primarily focusing on the impact of specific regional nodal doses, including SCN irradiation, on clinical outcomes.

## Conclusion

This mid-trial analysis provided insights into inter-institutional dosimetric deviations within the KROG 19 − 09 prospective cohort study. Significant deviations for regional nodal doses were observed in both the WBI + RNI and WBI groups, suggesting that current group allocation may not be appropriate to assess the clinical impact of RNI omission. Consequently, planning approaches were refined through institutional collaboration and an AI auto-contouring engine. Furthermore, final evaluation strategies will incorporate investigations into the association between specific nodal doses and clinical outcomes. We will also assess the AI auto-contouring engine’s role in reducing inter-institutional dosimetric deviations. These efforts are expected to enhance the overall quality and clinical interpretability of KROG 19 − 09.

## Supplementary Information

Below is the link to the electronic supplementary material.

Supplementary Material 1

Supplementary Material 2

Supplementary Material 3

## Data Availability

The datasets used and/or analysed during the current study are available from the corresponding author on reasonable request.
