# Supplementary material for: Mid-trial dosimetric analysis of the korean radiation oncology group 19 − 09 multi-institutional prospective cohort study on breast cancer
Source: Radiat Oncol. 2026 Aug 11;21:154. doi: 10.1186/s13014-026-02904-y (PMC13746673; doi:10.1186/s13014-026-02904-y)
Supplement: Supplementary file 1 [file 13014_2026_2904_MOESM3_ESM.docx]

Additional File 3. Counts for target under-coverage cases (D95% < 90% of prescribed dose) by physician in WBI+RNI group

| WBI+RNI case | | **Axl. level I** | **Axl. level II** | **Axl. level III** | **Interpectoral LN** | **SCN (ESTRO)** | **SCN (RTOG)** | **IMN** |  |
| --- | --- | --- | --- | --- | --- | --- | --- | --- | --- |
| **Sum** | **N=176** | 117 (66%) | 54 (31%) | 53 (30%) | 58 (33%) | 83 (47%) | 113 (64%) | 172 (98%) |  |
| **A** | **N=33** | 13 (39%) |  |  | 1 (3%) |  | 5 (15%) | 33 (100%) |  |
| **B** | **N=17** | 7 (41%) | 5 (29%) | 1 (6%) | 5 (29%) | 6 (35%) | 17 (100%) | 17 (100%) |  |
| **C** | **N=12** | 12 (100%) | 12 (100%) | 12 (100%) | 12 (100%) | 12 (100%) | 12 (100%) | 12 (100%) |  |
| **D** | **N=49** | 46 (94%) | 31 (63%) | 31 (63%) | 32 (65%) | 47 (96%) | 49 (100%) | 49 (100%) |  |
| **E** | **N=29** | 23 (79%) | 1 (3%) | 2 (7%) | 2 (7%) | 2 (7%) | 2 (7%) | 25 (86%) |  |
| **F** | **N=14** | 8 (57%) | 5 (36%) | 2 (14%) | 3 (21%) | 2 (14%) | 14 (100%) | 14 (100%) |  |
| **G** | **N=9** |  |  | 2 (22%) | 3 (33%) | 3 (33%) | 3 (33%) | 9 (100%) |  |
| **H** | **N=9** | 4 (44%) |  | 2 (22%) |  | 9 (100%) | 9 (100%) | 9 (100%) |  |
| **I** | **N=3** | 2 (67%) |  |  |  | 1 (33%) | 1 (33%) | 3 (100%) |  |
| **J** | **N=1** | 1 (100%) |  | 1 (100%) |  | 1 (100%) | 1 (100%) | 1 (100%) |  |
| *Abbreviations:* D95%, minimum dose delivered to 95% volume of the structure relative to the prescribed dose; WBI, whole breast irradiation; RNI, regional nodal irradiation; Axl, axillary; LN, lymph node; SCN, supraclavicular node; ESTRO, European Society for Radiotherapy and Oncology; RTOG, Radiation Therapy Oncology Group; IMN, internal mammary node | | | | | | | | | |
